# Comparison of Laboratory Data of Acute Cholangitis Patients Treated with or without Immunosuppressive Drugs

**DOI:** 10.1155/2014/619628

**Published:** 2014-03-10

**Authors:** Minoru Tomizawa, Fuminobu Shinozaki, Rumiko Hasegawa, Yoshinori Shirai, Noboru Ichiki, Yasufumi Motoyoshi, Takao Sugiyama, Shigenori Yamamoto, Makoto Sueishi

**Affiliations:** ^1^Department of Gastroenterology, National Hospital Organization Shimoshizu Hospital, 934-5 Shikawatashi, Yotsukaido City, Chiba 284-0003, Japan; ^2^Department of Radiology, National Hospital Organization Shimoshizu Hospital, 934-5 Shikawatashi, Yotsukaido City, Chiba 284-0003, Japan; ^3^Department of Surgery, National Hospital Organization Shimoshizu Hospital, 934-5 Shikawatashi, Yotsukaido City, Chiba 284-0003, Japan; ^4^Department of Neurology, National Hospital Organization Shimoshizu Hospital, 934-5 Shikawatashi, Yotsukaido City, Chiba 284-0003, Japan; ^5^Department of Rheumatology, National Hospital Organization Shimoshizu Hospital, 934-5 Shikawatashi, Yotsukaido City, Chiba 284-0003, Japan; ^6^Department of Pediatrics, National Hospital Organization Shimoshizu Hospital, 934-5 Shikawatashi, Yotsukaido City, Chiba 284-0003, Japan

## Abstract

*Objective.* Symptoms and laboratory data between acute cholangitis (AC) patients treated with and AC patients treated without immunosuppressive drugs (corticosteroids or methotrexate) were compared to identify factors that can be meaningful to the diagnosis of AC. *Methods.* The Wilcoxon signed-rank test was used for comparison of baseline variables between the patients with AC treated with immunosuppressive drugs and those without it. The chi-squared test was used in the analysis of the symptoms. *Results.* In total, 69 patients with AC were enrolled. Fifteen patients were treated with immunosuppressants due to rheumatoid arthritis or other collagen diseases. Jaundice was less frequent in the patients treated with immunosuppressive drugs (*P* = 0.0351). T-Bil level was marginally lower in the patients treated with immunosuppressants (*P* = 0.086). AST and ALT levels were lower in the patients treated with immunosuppressants (*P* = 0.0417 and 0.022, respectively). *Conclusions.* The frequency of jaundice and AST and ALT levels were lower in the patients treated with immunosuppressive drugs. It is recommended that care be taken to evaluate jaundice, AST level, and ALT level in the diagnosis of AC.

## 1. Introduction

Acute cholangitis (AC) is a bacterial infection caused by obstruction of the bile duct [1–3]. AC should be treated promptly because it can be fatal owing to sepsis [4, 5]. Biliary drainage is performed by endoscopic retrograde cholangiopancreatography (ERCP), percutaneous transhepatic cholangiography, or endoscopy-guided ultrasonography [6]. The prompt and accurate diagnosis of AC is a necessity. The diagnosis of AC is based on the presence of inflammation and biliary obstruction [7, 8]. Laboratory data are indispensable for the diagnosis of AC. These include the following: white blood cell (WBC) count and C-reactive protein (CRP), total bilirubin (T-Bil), alkaline phosphatase (ALP), aspartate aminotransferase (AST), alanine aminotransferase (ALT), and gamma-glutamyl transpeptidase (*γ*-GTP) levels [6, 9].

Some AC patients are simultaneously being treated for a collagen disease such as rheumatoid arthritis [10]. Collagen diseases are treated with immunosuppressive drugs that consist of corticosteroids, methotrexate (MTX), and, recently, biological agents such as etanercept. Corticosteroids include prednisolone and methylprednisolone. AST and ALT levels increase subsequent to the administration of a combination of corticosteroids and other immunosuppressive drugs [11]. The distribution of changes is not different between immunosuppressants. MTX antagonizes folate and inhibits DNA synthesis. It is also associated with hepatotoxicity [12]. Etanercept antagonizes tumor necrosis factor-*α* and suppresses the immune system [13]. This agent is also associated with hepatotoxicity [14]. Hepatotoxicity can potentially interfere with the correct diagnosis of AC. The alteration of WBC count and T-Bil levels may result in a failure to correctly assess the severity of AC [15]. A comparison of laboratory data between the AC patients treated with and those treated without immunosuppressive drugs is, however, not available.

We therefore compared laboratory data between AC patients treated with and AC patients treated without immunosuppressive drugs.

## 2. Materials and Methods

### 2.1. Inclusion Criteria

Patient records from April 2008 to March 2013 were retrospectively analyzed. Our study was subjected to approval by our institutional ethical committee and determined not to be a clinical trial since it was performed as part of daily clinical practice. Written informed consent was obtained for each session of ERCP. Written informed consent to undergo contrast-enhanced computed tomography (CECT) or magnetic resonance cholangiopancreatography (MRCP) was also obtained from the patients. Patient anonymity was preserved.

### 2.2. Immunosuppressive Drugs

The immunosuppressants used included prednisolone, methylprednisolone, and MTX. Biological agents such as etanercept were also included.

### 2.3. Diagnostic Criteria for Acute Cholangitis

The patients were diagnosed with AC when they showed fever, abdominal pain, and jaundice (Charcot's triad). If a patient did not show all the components of Charcot's triad, AC was diagnosed in the presence of an inflammatory response and biliary obstruction. An inflammatory response consisted of fever, elevation of WBC count, or elevation of C-reactive protein level. Biliary obstruction consisted of bile duct dilatation, biliary stricture, a common bile duct stone, ALP level elevation, or *γ*-GTP level elevation. The severity of AC was assessed according to the Tokyo Guidelines (TG13) [8]. Patients were considered to have severe AC when they showed at least one of the following: cardiovascular, neurological, respiratory, renal, hepatic, or hematological dysfunction. Moderate AC was defined as the presence of at least 2 of the following abnormalities: abnormal WBC count, high fever, high T-Bil level, and hypoalbuminemia.

### 2.4. Endoscopic Retrograde Cholangiopancreatography

ERCP procedures were performed by experienced endoscopists with JF-260V video duodenoscopes (Olympus, Tokyo, Japan). Papillotomies were performed with a pull-type sphincterotome (Boston Scientific, Natick, MA). Stones or sludge were removed with a basket or balloon catheter. If necessary, a nasobiliary catheter was inserted for drainage.

### 2.5. Imaging Diagnostics

Patients with suspected AC underwent CECT and abdominal ultrasound to further investigate biliary dilatation, common bile duct stones, and cancer. From May 2012, the patients underwent MRCP using a 1.5-Tesla scanner (Achieva, software version 3.2.2, Philips Medical Systems, Best, The Netherlands). Before May 2012, some of the patients were referred to Sannou Hospital (Chiba City, Japan) for MRCP. CECT was performed using a 16-detector row CT scanner (SOMATOM Emotion 16, Siemens, Munich, Germany). The contrast medium was administered intravenously as follows: 100 mL of iopamidol at 3 mL/s (Konica Minolta Healthcare, Tokyo, Japan). CT images were acquired before the injection of contrast medium, and 30, 70, and 180 s later. Abdominal ultrasound was performed with an SSA-700A instrument (Toshiba Medical Systems Corporation, Ohtawara, Japan) by senior fellows of the Japan Society of Ultrasonics in Medicine, using a 5.0 MHz curved-array transcutaneous probe or an 8.0 MHz linear-array transcutaneous probe.

### 2.6. Statistical Analysis

The Wilcoxon signed-rank test was used for comparison of baseline variables between the AC patients treated with and those treated without immunosuppressive drugs. The chi-square test was used in the analysis of the symptoms and severity of AC.

## 3. Results

In total, 69 patients with AC were enrolled. Thirty-seven were male (mean ± SD age, 69.5 ± 8.3 years), and 32 were female (mean ± SD age, 68.2 ± 12.3 years). AC was caused by bile duct stones in 66 cases, bile duct cancer in 2 cases, and pancreatic cancer in 1 case. Eight patients were treated with a corticosteroid, and 4 were treated with MTX (Table 1). Three patients were treated with a combination of a corticosteroid and MTX. Fourteen patients were treated with immunosuppressive drugs for rheumatoid arthritis.

Symptoms are important for the diagnosis of AC. Symptoms were compared between the patients who were treated with immunosuppressive drugs and those who were not.  Table 2 shows a comparison of the number of patients with abdominal pain, fever, and jaundice in each group. The number of patients with jaundice was significantly lower among those treated with immunosuppressive drugs (*P* = 0.0351).

Blood examination results were also compared between the 2 groups of patients (Table 3). The WBC count was marginally higher in the patients treated with immunosuppressive drugs than in those who were not. AST and ALT levels were significantly lower in the patients treated with immunosuppressants.

Finally, the severity of AC was compared between the 2 patient groups (Table 4). The percentage of mild, moderate, or severe AC did not differ significantly between the 2 groups.

## 4. Discussion

Overall, no significant differences in laboratory data were observed between the patients treated with and those treated without immunosuppressive drugs. Abdominal pain is omitted from the TG13, but the symptom is still important [8]. Fever is an indicator of inflammation. Our study shows that the presence of both symptoms is similar between the AC patients treated with and those treated without immunosuppressive drugs. This suggests that the diagnosis of AC can be expected to be made with similar accuracy in both patient groups.

Jaundice is a component symptom in Charcot's triad. In our study, the frequency of jaundice was lower in the patients treated with immunosuppressants. T-Bil level was marginally lower in the patients treated with immunosuppressive drugs. Consistent with our results, corticosteroids were shown to reduce T-Bil in patients with biliary atresia [16]. This report and our data suggest that immunosuppressive drugs decrease T-Bil levels. However, the mechanism of this reduction is not known. With regard to AC, it is recommended that jaundice and T-Bil level be carefully evaluated during diagnosis.

Unexpectedly, AST and ALT levels were lower in the patients treated with immunosuppressive drugs. These were expected to be higher in the patients treated with immunosuppressive agents because they potentially cause hepatotoxicity. The reason is not known. It is speculated that the mechanism of hepatotoxicity differs between immunosuppressant drugs and biliary obstruction. Drug-related hepatotoxicity comprises drug-induced liver injury and is associated with apoptosis [17]. Conversely, bile duct obstruction causes damage to hepatocyte membranes via bile acids, accumulated copper, and membrane peroxidation [18, 19].

One might expect that immunosuppressive agents could be applied to AC patients to reduce the damage of liver caused by obstructive jaundice. The patients should be treated with ERCP and the other intervention [6]. The elevated liver damage would be decreased.

In conclusion, it is recommended that care be taken to avoid underestimating AST and ALT levels for the diagnosis of AC according to the TG13 [8].

## 5. Conclusions

The frequency of jaundice and AST and ALT levels were lower in the patients treated with immunosuppressive drugs. It is recommended that care be taken to evaluate jaundice and AST and ALT levels in the diagnosis of AC.

## Figures and Tables

**Table 1:** Patients' characteristics.

	Male	Female	Total
Number	37	32	69
Age, years	69.5 ± 8.4	65.3 ± 12.3	67.5 ± 10.5
Cause of acute cholangitis			
Bile duct stone	35	31	66
Bile duct cancer	2	0	2
Pancreatic cancer	0	1	1
Immunosuppressant			
(−)	33	21	54
Corticosteroid	1	7	8^a,b^
Methotrexate	2	2	4^c^
Corticosteroid + methotrexate	1	2	3
Immunosuppressant indication			
Rheumatoid arthritis	3	11	14
Microscopic polyangitis	1	0	1
Polyarteritis nodosa	0	1	1
Polymyalgia rheumatica	0	1	1

^a^One female with methylprednisolone, and the other patients with prednisolone; ^b^one female with prednisolone and etanercept; ^c^one female with methotrexate and etanercept.

**Table 2:** Comparison of symptoms.

	Abdominal pain (*P* = 0.6315)		Total	Fever (*P* = 0.6293)		Total	Jaundice (*P* = 0.0351)		Total
	(−)	(+)		(−)	(+)		(−)	(+)	
Immunosuppressant									
(−)	18	36	54	29	25	54	23	31	54
(+)	6	9	15	7	8	15	11	4	15

Total	24	45	69	36	33	69	34	35	69

The *P* values indicate the statistical significance according to the chi-square test.

**Table 3:** Comparison of patient baseline variables.

	Immunosuppressant (−)		Immunosuppressant (+)		*P* value
	Average	95% CI	Average	95% CI	
WBC	8,802	7,473–10,130	11,506	8,985–14,027	0.0735
CRP	4.96	2.93–7.00	6.74	2.92–10.56	0.9646
T-Bil	2.50	1.61–3.39	2.24	0.55–3.93	0.0860
ALP	661	537–784	629	399–859	0.6249
AST	182	117–247	176	53–300	0.0417
ALT	200	147–254	148	45–252	0.0220
*γ*-GTP	380	287–473	339	165–512	0.3395

WBC: white blood cell; CRP: C-reactive protein; T-Bil: total bilirubin; ALP: alkaline phosphatase; AST: aspartate aminotransferase; ALT: alanine aminotransferase; *γ*-GTP: gamma-glutamyl transpeptidase; CI: confidence interval.

**Table 4:** Comparison of acute cholangitis severity.

		Severity (*P* = 0.9694)			Total
		Mild	Moderate	Severe	
Immunosuppressant					
(−)		44	6	4	54
(+)		12	2	1	15

Total		56	8	5	69

The *P* values indicate the statistical significance according to the chi-square test.
